# Restoration of motor function after CNS damage: is there a potential beyond spontaneous recovery?

**DOI:** 10.1093/braincomms/fcab171

**Published:** 2021-07-30

**Authors:** Volker Dietz

**Affiliations:** Spinal Cord Injury Center, University Hospital Balgrist, Zürich, Switzerland

**Keywords:** CNS damage, motor function, neuroplasticity, neurorehabilitation

## Abstract

What determines the effectiveness of neurorehabilitation approaches on the outcome of function in stroke or spinal cord injured subjects? Many studies claim that an improvement of function is based on the intensity of training, while some actual studies indicate no additional gain in function by a more intensive training after a stroke. Inherent factors seem to determine outcome, such as damage of specific tracts in stroke and level of lesion in spinal cord injured subjects, while the improvement of function achieved by an intensive training is small in relation to the spontaneous recovery. It is argued that an individual capacity of recovery exists depending on such factors. This capacity can be exploited by a repetitive execution of functional movements (supported as far as required), irrespective of the intensity and technology applied. Elderly subjects have difficulties to translate the recovery of motor deficit into function. Alternative, non-training approaches to restore motor function, such as epidural or deep brain stimulation as well as CNS repair are still in an early clinical or in a translational stage.

## Teaser

Inherent factors determine outcome of function after a CNS lesion, i.e. damage of specific tracts in stroke and level of lesion in SCI subjects. There exists an individual capacity of recovery. This capacity can be exploited by a repetitive execution of functional movements irrespective of the intensity or technology applied.

## Graphical Abstract

**Graphical Abstract:**
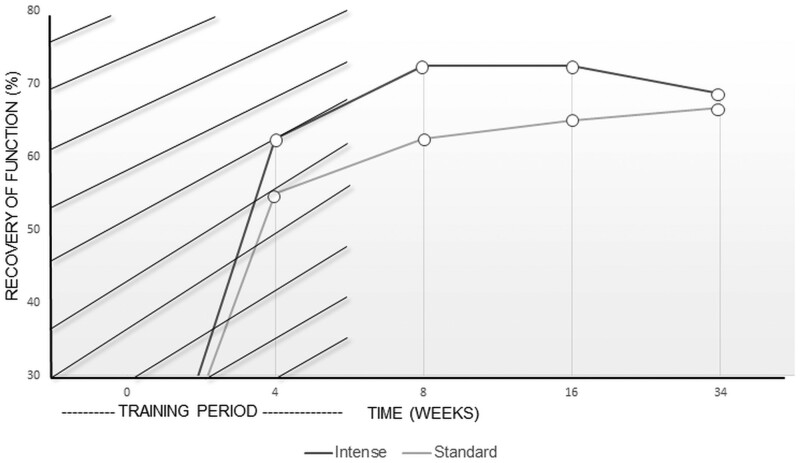

## The issue: What is the impact of neurorehabilitation on the recovery of function?

The number of patients suffering a CNS damage, most frequently due to a stroke, increase continuously due to an ageing population. Most of these patients undergo neurorehabilitation procedures. There is no standard treatment of motor deficits after a CNS damage, i.e. the procedures applied differ between countries and centres within the same country. The effectiveness or superiority of any rehabilitation approach can hardly be demonstrated on a strong scientific basis as ethical issues do not allow comparison between treated and non-treated patients and robust studies which compare different approaches or demonstrate best evidence for an intervention are rare.^1^

Most rehabilitation approaches are believed to achieve a gain in function by the exploitation of neuroplasticity.^1–3^ The success of this exploitation is suggested to be influenced by a number of factors, such as age or severity of CNS damage^4^ or, e.g. by task complexity.^5^ In the neurorehabilitation of stroke or SCI subjects, the idea of exploitation of neuroplasticity becomes usually implemented in rehabilitation by the repetitive execution of functional movements of impaired limb(s), requiring synergistic muscle activation,^6^ i.e. for example, reach and grasp movements for upper extremities and stepping movements for lower limbs. The training can be complemented by passive muscle stretching when a deforming spastic paresis is present.^7^^,^^8^ In stroke and SCI subjects by such an approach some recovery of function is usually achieved, even in elderly subjects,^9^ with a maximum within about three to four months after CNS damage.^10^ However, outcome of function not only depends on training but also on other factors, such as infections which are known to impair recovery of function.^11^

A more intensive movement training was suggested to lead to a better outcome of function.^12^^,^^13^ As a consequence, technology entered the field of neurorehabilitation. Robotic devices became developed that allowed longer training times in combination with monitoring changes in function.^12^^,^^14^ During the last 25 years, a large number of such devices came into the market with the aim to achieve greater rehabilitation effects by an optimal exploitation of neuroplasticity by a higher number of movement repetitions.^14^^,^^15^

The question underlying this review is whether an additional, substantial gain of function can be gathered by a high intensity training in relation to the recovery of function achieved by a standard training. This aspect is related to the question in how far an improvement of function occurs to a large extent spontaneously or is, alternatively, due to specific rehabilitation approaches. The term ‘spontaneous’ in the present context is considered as the recovery of function following a regular, (i.e. several times/day), repetitive execution of upper/lower limb movements used in daily life activities with a personal/technical support required. Without the performance of such movements, i.e. when limbs remain immobilized due to the paresis, little spontaneous recovery is expected to occur. Instead, muscle/joints contractures will develop—similar as in conditions with limb immobilization due to other causes than CNS damage, e.g. bone fractures. The capacity of functional recovery is suggested to consist in a combination of resolving neurapraxia and neuroplasticity.

On the basis of actual scientific achievements and discussions in the field,^14^^,^^16^^,^^17^ it is argued that most part of functional recovery occurs in so far ‘spontaneously’, as it is determined by the exploitation of an individually limited capacity for a recovery. Furthermore, it is suggested that the success of this exploitation does not depend on specific rehabilitation interventions.^16^^,^^17^

## Inherent factors determining outcome of function

*After a brain damage* that includes pyramidal tract connections to hands and fingers the motor deficit can hardly be compensated by the activation of other non-damaged tracts/brain areas.^18–20^ As a result, a quite limited recovery (10–20%) of paralyzed fingers occurs.^21^^,^^22^ The minor signs of recovery have been suggested to occur spontaneously (e.g. resolving neurapraxia?), i.e. without evidence for training effects.^23^ In contrast, following damage of other brain areas a more favourable recovery of function of proximal arm and leg muscles can be expected (60–80%; Table 1).^18–20^ This is achieved by a standard therapy, i.e. the repetitive execution of functional movements over a limited time (e.g. 30–50  min per day) which become supported as far as needed.^24^

**Table 1:** Main aspects of neurorehabilitation and outcome of upper limbs following stroke or cervical SCI

	Location	Typical recovery course	Goal	Rehabilitation approach
Stroke	Damaged Corticospinal tract (CST)	Little recovery, esp. chronic impairment of hand/finger extension	Prox. arm muscle activation; avoidance of muscle contractures; use of impaired limb for support/holding function	Prox. arm muscle strengthening; repetitive passive limb motion; training of compensatory strategies
	Intact CST	Spontaneous recovery of ∼70% of initial arm/hand impairment	Arm and simple grasping function; uni-/bimanual ADL functions	Functional reach/grasp and bimanual (cooperative) hand movements; strengthening of wrist/finger extensors; simple movement training with transfer to ADL; limited dose-dependent training effects
SCI	Lesion level C6/7	Spasctic forearm flexor muscle tone impending the development of tenodesis grasp	Tenodesis grasp; bimanual grasp	

*After spinal cord damage*, the improvement of upper limb function depends on the level, and extent of lesion.^10^ In cervical cord injuries, a combined damage of central (spinal tracts) and peripheral nerval structures (motoneurons and roots to arm, hand and finger muscles) occurs. This results in an arm/hand/finger paresis associated with a mixture of spastic and flaccid muscle tone^25^ (Table 1). The peripheral part of nervous system damage can account for up to 50% of paresis.^26^ This part of nerval damage has little potential to recover. After a sensori-motor complete SCI any recovery of function is rather unlikely to occur.^24^

*The age* of patients has little influence on the recovery of the neurological deficit in post-stroke^27^^,^^28^ and SCI^29^ subjects, i.e. it is similar in elderly and young subjects. However, after an SCI young compared to elderly subjects can better translate the recovery of motor system deficits into functions required in daily life activities.^29^

It is concluded that there is an inherent, individual capacity of recovery of function after a stroke or SCI that depends on factors, such as location and severity of CNS damage. This capacity can be determined early after CNS damage by clinical, electrophysiological^24^ and imaging^19^ examinations. These measures can also be used as prognostic factors and, consequently, for the selection of appropriate rehabilitation procedures early after CNS damage.

## Compensatory role of spastic muscle tone

**Figure 1:**
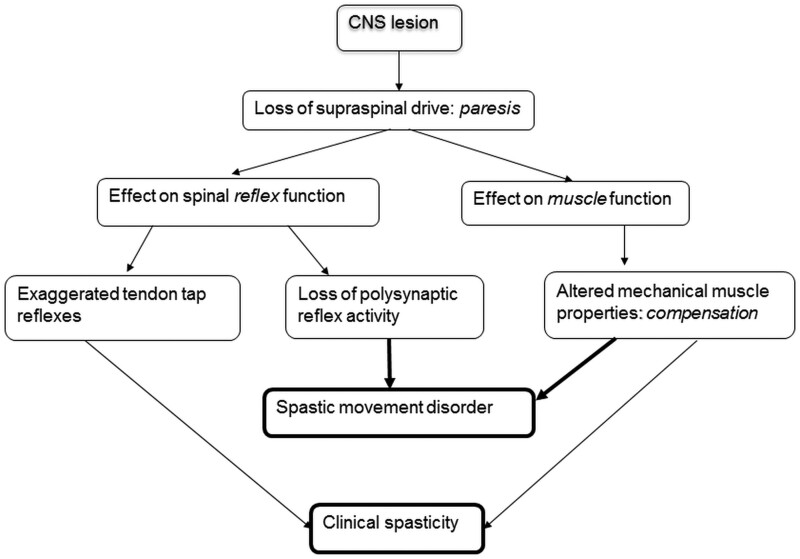
**Mechanisms leading to spastic movement performance.** A CNS lesions affecting motor behaviour leads to a loss of supraspinal drive. As a consequence, alterations of proprioceptive feedback, i.e. changes in the excitability of spinal reflexes and in muscle function, reflected in altered mechanical muscle properties, occur. The combination of all sequels of the primary lesion leads to the divergent appearance of clinical spasticity and spastic movement disorder. Modified after Dietz and Sinkjaer.^30^

After a stroke/incomplete SCI, a loss of supraspinal drive leads to a paresis and, consequently, reduced mobility. With the development of spastic muscle tone, this deficit becomes partially compensated (Fig. 1). Functional movements, such as stepping, can be executed on a lower level of organization.^30^ Therefore, most post-stroke subjects regain walking function by using the spastic-paretic leg more or less stick-like: Support of the body in the stance phase and circumduction of the leg during swing (due to reduced knee flexion). The normal push-off the leg at the end of stance phase is lost. As a consequence, the limited improvement of walking ability achieved over the course of rehabilitation after a stroke is associated with little change in biomechanical and muscle activation characteristics of the spastic-paretic leg.^24^^,^^31^ The improvement in mobility is, therefore, rather due to adaptational changes than due to a restoration of ‘normal’ stepping function.

Patients suffering a cervical SCI (i.e. C6/7) require spastic muscle tone to perform simple grasp movement (the so-called tenodesis grasp). Furthermore, spastic proximal arm muscles can provide some passive gravity support to carry an object from one to another spot (cf Table 1).

## More intensive training: More gain in function?

Several studies indicate that by a more intensive training an additional gain in function of upper and lower limbs can be achieved. This effect was reported for post-stroke subjects,^1–3^^,^^32–35^ as well as for subacute^36^ and chronic^37^ SCI subjects.

However, in none of these studies the additional gain of function was related to the recovery of function achieved by a standard training or to the spontaneous recovery of function. In fact, for lower limb function the improvement of outcome achieved by a more intensive training is small (or transient; cf. fig. 3 of Hubli et al.^38^) in relation to the gain in function achieved by a standard training in post-stroke^38^ and SCI^36^ subjects. For upper limb function of chronic post-stroke subjects, there is no evidence for a dose–response effect of training intensity on functional recovery.^39^

Can a more intensive locomotor training improve stepping function after a stroke? In a large group (200 adults) of moderately to severely impaired subacute post-stroke subjects, a bodyweight supported treadmill training was not superior to relaxation sessions (of same duration and in addition to standard therapy) in respect of walking speed and activities of daily living (ADL).^16^ Correspondingly, in incomplete SCI subjects doubling of the daily locomotor training time had only small effects on walking ability.^36^

## Alternative non-training approaches to restore motor function

### SCI repair

What is the best cell candidate for a transplantation-based treatment of brain or pinal cord injury and, which kind of CNS damage should preferentially be treated? These issues remain an ongoing matter of investigations.^40^ Application of Schwann cells,^41^ stem cells^42^ or auto-transplantation of olfactory ensheathing cells:^43^ All these cell types are known to be permissive for the outgrowth of lesioned spinal or supraspinal tract axons in animal models of CNS damage.

In the case of the transplantation of olfactory ensheathing cells in SCI subjects, neither negative nor beneficial effects were found in individuals with motor complete spinal cord injury.^43^ The same is true for transplantation of foetal stem cells in China^44^ and for the application of human neural stem cells in cervical SCI^45^ which both did not show signs of motor recovery. Besides cell-based repair, the application of Nogo-antibodies was shown to be effective for SCI repair in animal experiments.^46^^,^^47^ A Nogo-antibody treatment is currently applied in patients suffering a cervical SCI in a phase two trial. If this treatment can successfully applied in human SCI it can be translated to the more complex condition of brain damage.

Treatment of cervical SCI aims to improve arm/hand function. The problem is that at the cervical level a combined damage of central and peripheral nervous structures exists. A thoracic spinal cord repair again would functionally be less important as at best a rudimentary stepping function could be achieved.

### Epidural spinal cord and deep brain stimulation

Epidural stimulation of spinal (thoraco-lumbar) neuronal networks facilitates the performance of stepping movements in SCI individuals with spared descending connections. In combination with spastic muscle tone this stimulation approach enhances walking ability.^48^ The success of this approach is in so far limited as only rudimentary steps can be executed with the support of crutches to maintain body balance. This means that by this approach subject have problems to carry an object from one to another spot. As a consequence, for the execution of ADL activities a wheelchair travelling is more effective.

Also, deep brain stimulation was shown to improve motor function in rodents with CNS damage.^49^ This approach is on the way to be translated to human beings.

## Conclusions

The question underlying this review is in how far a more intensive training leads to an additional gain in function in relation to a standard training. The answer is that by an intensive training some additional recovery of function can be achieved. However, this gain in function is small, transient, or even can be absent in relation to the ‘spontaneous’ recovery of function. It is concluded that there is an *individually limited capacity of recovery of function* after a stroke or SCI that depends on inherent factors such as location and severity of CNS damage.

The improvement of function within this capacity depends on the appropriate activation of motoneuron pools of synergistic limb muscles under physiological movement conditions. This means, the exploitation of this capacity is based on the standard rehabilitation approach, i.e. the repetitive execution of functional movements (with the support of a therapist or a device as far as required). On this basis, the recovery of function is achieved irrespective of the rehabilitation intervention applied.

The recovery of a motor deficit after stroke or SCI occurs independent of age. However, in SCI subjects the gain in motor system capacity can better be translated into function in young compared to elderly subjects.

Considering these aspects, an integral part of rehabilitation should be directed to compensate the remaining motor deficit by refined assistive devices which allow a self-independent life as far as it is possible for the individual patient.

Alternative, training supplementary approaches, such as epidural or deep brain stimulation might somewhat enhance motor function, e.g. improve the ability to perform stepping movements in subjects suffering a CNS damage. A repair of the damaged spinal cord/brain is presently not yet available.

This review on the recovery of motor function after CNS damage has to be based on a rather limited scientific evidence present in the field of neurorehabilitation. More large scaled trials, including defined patient groups, are needed to definitively estimate the effect size of a more intensive training approach.

No new data were generated in the article.

## Search strategy and selection criteria

Original research papers were cited that included a sufficient number of patients and reviews devoted to original key studies on rehabilitation effects on outcome of motor function in stroke and spinal cord injured subjects published in high quality journals during the last 20 years.

## Competing interests

The authors report no competing interests. 

## Abbreviations

SCI =: spinal cord injury
CST =: corticospinal tract
